# Exploratory Study of Depressed Adolescents’ Life Narratives

**DOI:** 10.5334/pb.bh

**Published:** 2015-06-16

**Authors:** Aurore Boulard

**Affiliations:** 1 Département de psychologies et cliniques des systèmes humains, University of Liège, Belgium

**Keywords:** Adolescent, depression, depressive mood, life narratives, textual data analysis

## Abstract

**Objective:** The aim of this study was to explore the life stories of depressive adolescents and compare them with non-clinical adolescents’ life stories. **Methods:** For this purpose, we compared 20 life stories of hospitalized adolescents suffering from major depressive episode with 40 life stories of adolescents attending school divided into two groups: 20 non-depressed and 20 depressed adolescents. **Results:** Results showed that life stories differed as a function of psychopathology. Depressed hospitalized adolescents spoke about their disease and defined themselves by their depression. The depressed adolescents in school concentrated on schooling and school achievements, while the non-depressed group defined themselves by their family, friends and inclusion in a peer group. **Conclusion:** These analyses allowed us to highlight specific themes mentioned by each of the three groups of adolescents. Although life stories are personal and unique, analysis of such stories allows us to better understand the daily reality of depressive adolescents and the relationships between the life events they experience, daily stressors, depression and how they construct their personal history.

## Introduction

Depression is one of the most common psychopathologies in adolescence. The specific symptom of depressive mood is present in 30% to 40% of adolescents in regular school settings (Boulard et al., 2012). These results support Twenge and Nolen-Hoeksema’s (2002) finding that adolescence is a sensitive period for the development of depression and depressive mood. In adolescence, the rates for major depressive episode (MDE) vary from 5% to 7%, with a prevalence of two girls for every boy (Alvin & Marcelli, 2005). According to Ustun et al. (2004), two-thirds of adolescents do not receive medical and/or psychological treatment. This lack of treatment can be explained by deficiencies in primary care (e.g., lack of knowledge regarding the identification and treatment of depression leading to under-diagnosis, under-estimation of the level of severity of the disorder, etc.) especially during adolescence when the symptoms of depression overlap with the “unhappiness” characteristic of this stage of development (Marcotte, 1995). The most commonly cited risk factors include negative life events, the onset of puberty, parental attachment, attachment to friends, school context, etc.

One of the effects of depression that has long been recognized is that it affects speech patterns (Mundt et al., 2007). Researchers have found changes in pitch, fluency and prosody, and articulation (Darby & Hollien, 1977; Alpert, Pouget, & Silva, 2001). Discourse features have also been highlighted. The first studies of depressed patients’ ways of thinking conducted by Beck (1976) showed that they reported more negative events when they talked about their past. According to Coyne and Gotlib (1983), depressed people are more likely than others to make internal attributions regarding these negative life events. They project their feelings of powerlessness onto the world (Seligman, 1975). This excessive attribution of responsibility should translate into significant use of the personal pronoun “I” (Mirabel-Sarron, 1992). Bucci and Freedman (1981) also observed that patients with depression used the pronoun “I” more frequently than other people and spoke of past events more often than patients in their control group. The experience of time is altered in depression: time is experienced as “stopped,” without a future, and with the past emerging from the present (Kuhs et al., 1991). That is why depressed patients are sometimes described as living in the past (Habermas et al., 2008).

Many articles on life stories have been published in recent years (McAdams & Pratt, 2006; McLean, Pasupathi, & Pals, 2007; McAdams, 2008). Their place in the field of personality psychology has been examined (Thorne, 2000; Bamberg, 2004; Fivush, 2008), as have the development and emergence of the ability to construct a coherent life story (Habermas & Bluck, 2000). However, few researchers have studied both the structure and content of narrative with depressed adolescents.

An argument for the role of life narratives in psychopathology holds that they not only evoke and reflect psychopathological mechanisms, but also reflect and perpetuate essential characteristics of the self (Habermas et al., 2008). Using words, a particular sentence structure, or a particular grammar, more or less consciously, the individual registers his or her story in a personal context. According to Habermas et al. (2008) both the form and the content of life stories depict essential aspects of the self and show how individuals integrate the experiences they have had into a coherent whole. The discourse analysis proposed in this research is based on the idea that there are connections between the linguistic system and the cognitive system. In Fallery and Rhodain’s (2007) view, discourse analysis supports both aspects of referential coherence (what the text refers to: nouns, linguistic signs that refer to extralinguistic reality) and those relating to the context of utterance (verbs, adverbs, conjunctions, connectors, etc., used to reflect speakers’ relationship to the situation, their views and judgments). The analysis of depressed patients’ discourse is often reduced to psychotherapeutic speech to evaluate treatment (Viederman & Perry, 1980). Some researchers have studied the timbre of the voice (Low et al., 2011) and its breaks (Greden & Carroll, 1980), and even detected depression from voice characteristics (Asgari, Shafran, & Sheeber, 2014). However, depressed adolescents’ discourse has been rarely analyzed. Yet determining how adolescents organize major events that cause stress to them is an essential process in clinical psychology and psychotherapy.

The purpose of this exploratory research is to highlight the specificities both in form and in content of the life stories of depressed adolescents in a clinical setting and to compare them with the life stories told by teenagers attending school who score high for depression. These first results will allow us to make assumptions about the continuity of depressive symptomatology. Indeed, researchers today agree that the depressive phenomenon is a continuous process and not a categorical one. Thus, the comparison of three groups (control, depressive at school and hospitalized) will allow us to make clinical hypotheses regarding this assumption of continuity. Several researchers (Eysenck, Wakefield, & Friedman, 1983; Widiger & Trull, 2007) have argued that certain disorders, if not all, can be seen as the end of a continuum from normal to pathological (Ruscio & Ruscio, 2000). This is particularly true of depression, which is seen on the one hand as a distinct disease (categorical) with a specific diagnosis (e.g., DSM-5), but also as a quantitative deviation from a normal emotional experience (Solomon, Haaga, & Arnow, 2001). Flett, Vredenburg, and Krames (1997) showed that one way of conceptualizing the continuum of depressive symptomatology is to identify individuals with subclinical depressive symptoms and determine when these symptoms result in a risk of developing major depression later on. In sum, how can depressive mood predict the development of severe depression in the years to come? Gotlib, Lewinsohn, and Seeley (1995) concluded that depressive mood is a risk factor for adolescents. It is therefore important to consider clinical significance alone (Compas, Orosan, & Grant, 1993). The results of Hankin et al. (2005) also point in this direction.

To compare the stories of groups of adolescents, we chose to apply statistical methods to the study of texts; this method constitutes an extremely rich exploratory approach both in the comparative study of texts and in the understanding of their content (Guérin-Pace, 1997). The advantage of this method is the ability to combine qualitative and quantitative results.

Although this study is exploratory, we made two hypotheses. The first was that the pronoun “I” would be used particularly frequently in the life narratives of depressed adolescents. The second was that depressed adolescents, and specifically adolescents hospitalized for depression, would talk more about negative life events in their stories.

## Methods

### Participants

Our sample was composed of 60 adolescents aged 12 to 18, in three different educational streams in the French Community of Belgium. This sample was divided into three groups:

- Group 1 (non-depressed adolescents attending school) consisted of 20 normally schooled adolescents. They obtained scores of less than 24 on the CES-D (see below).
- Group 2 (depressive adolescents attending school) consisted of 20 adolescent students who scored more than 24 on the CES-D.
- Group 3 (depressed hospitalized adolescents) was composed of 20 adolescent inpatients in a child psychiatry unit for whom a diagnosis of major depressive episode had been made by a child psychiatrist. Patients with a comorbid drug addiction were excluded. These adolescents were hospitalized in the short-stay section of a child psychiatry department. They had never been hospitalized before. The diagnosis of depression was made for the first time. It should be noted that the age limit for hospitalization in child psychiatry is 18 years. We therefore chose to limit the study to this age period.

The hospitalized group of depressive teenagers served as the basis for pairing all subjects by sex, age and educational stream. Each group included 13 girls and 7 boys divided into three different educational streams: 9 teenagers from the general education stream, 8 from professional education and 3 from technical education. They had a mean age of 15 years (minimum 12 years and maximum 18 years). The overall sample was composed of 39 girls (65% of the sample) and 21 boys, with 45% from the general education stream and 55% from the other two streams combined.

It should be noted that, in order to pair hospitalized adolescents with adolescents in school (depressed or not), we collected life stories from 126 adolescents from different educational streams. According to Hammen and Rudolph (2003), 15% to 30% of adolescents suffer from depression. In this group, 40 teenagers obtained scores above 24 on the CES-D and 88 teenagers scored below the cut-off score.

### Procedure

First, the research was explained to the parents of all the adolescents. They were contacted by letter with the authorization of the school principal (for groups 1 and 2) and directly in the case of the depressed hospitalized adolescents. After the parents gave their informed consent, we met the young people and described our research to them. One hundred and twenty-six adolescents attending school and 20 depressed hospitalized adolescents consented to participate in our study. Three adolescents who were hospitalized for depression chose not to participate. The average agreement rate of adolescents attending school to participate in our study was 25%.

We personally met and interviewed the 20 hospitalized adolescents. They were interviewed in a private hospital room in the child psychiatry unit. The other 126 participants were interviewed during their lunchtime at school, in a classroom. All adolescents were seen alone. They were interviewed by the author and three psychology students trained to conduct this type of interview. The interview took about 50 minutes and was divided into two parts. The first part of the interview consisted of the administration of several scales including a socio-demographic questionnaire and the CES-D. The questionnaires were not examined in front of the teenagers. The second part was dedicated to the adolescent’s life story.

### Material

**CES-D.** The Center for Epidemiologic Studies Depression Scale (CES-D; Radloff, 1977) is a self-administered questionnaire with 20 items. Each response is scored from 0 to 3. This scale has been shown to measure depressive symptoms in the general population. Its validity has been confirmed in many studies among student communities, as well as among adults and adolescents in other settings (Chabrol et al., 2002; Boulard, Gauthier, & Born, 2014). Scores range from 0 to 60 with a cut-off score of 24. With this threshold score, sensitivity of 0.74 and specificity of 0.73 are obtained. The results on the CES-D can be categorized in four classes: 0–9 = no or minimal depression, 10–16 = mild depression, 17–24 = moderate depression, and 24 = moderate to severe depression.

**Life narratives.** Participants were asked to tell their life stories. The question they were asked was: “How did you become what you are today?” If the adolescent did not understand the question, the researcher asked it in a different form: “Were there any positive or negative life events that made you become what you are today?” The researcher let the teenagers build their own life stories, providing nonverbal encouragement when they needed it.

Each life story was recorded and then transcribed by the researcher who had administered the interview. The recording time was standardized and set at 10 minutes (after a pre-test done with normally schooled adolescents).

Each story was read and encoded by a single researcher to minimize transcription bias. It was decided that onomatopoeia and hesitations (incomplete words) would not be taken into account because the purpose of this analysis was to highlight the morpholexical structure of speech. We did not take meta-information (facial expressions, body position, and intonation) into account either. Our choice can be seen as a reduction of spoken discourse; however, even if one ignores the meta-information, the narration of a text is a particularly complex process: the words of the text relate to each other in ways that can be revealed by counts performed on the entire text (Lebart & Salem, 1994) or compared to other productions.

Each speech was transformed into a text file that contained meta-information such as the adolescent’s age, sex, name and membership in one of the three groups (clinically depressed and hospitalized, depressive and at school, and non-depressed and at school).

## Results

To conduct our analyses, we used two different textual data analysis software applications (Lexico3 and Hyperbase). The use of this software allowed us to take many life stories into account and process the data using advanced statistical analysis methods. The corpus was previously labeled with Cordial® syntactic annotation software (Synapse Development Society). Syntactic labeling of a text is a fundamental step in the analysis, and a preliminary to any automatic processing at the highest level (Eshkol et al., 2001). The purpose of labeling is to assign each word in a corpus a label that summarizes its morphosyntactic information. The Cordial Analyzer version allows both the labeling of texts and the statistical processing of labeled texts. The labeling is very complete. The program recognizes 201 grammatical types, lemmas,^1^ grammatical functions of words and different kinds of semantic information (Poudat, 2004).

### Descriptive Statistics

As a control, we first calculated the mean scores on the CES-D for each of our three groups. The mean score for the non-depressed adolescents in school is 9.4 (min = 5.5, max = 13.3). For the group of depressed adolescents attending school, the mean is 33.1 (min = 29.2, max = 36.7), and for the group of hospitalized adolescents, it is 34.1 (min = 30.2, max = 37.9). A one-way ANOVA (*F* (57, 2) = 52.25, *p* < .000, adjusted R^2^ = .63) showed a significant difference between the three groups. The post hoc test highlighted a significant difference (*p* < .05) between the non-depressed group of adolescents and the other two groups. There were no differences between the two groups of depressed adolescents (school and hospital).

### Main Lexicometric Characteristics

The corpus of 60 life stories of is composed of 53,282 words and 9,293 lexical forms (Table 1).

**Table 1:** Lexical statistics for the three groups of adolescents.

Group	Words	Lemmas

Non-depressed adolescents attending school	24,721	2,023
Depressed adolescents attending school	23,990	1,969
Depressed adolescents in hospital	14,061	1,584
Total	62,772	3,490^*^

^*^This is not the total sum of the lemmas but the total sum of different lemmas in the corpus; a lemma in one class can also be found in another, which is why it is not a case of simple addition.

A one-way ANOVA with the number of words as the independent variable showed a significant difference between the three groups (*F* (2,56) = 6.74, *p* < .01; adjusted R^2^ = .17). Post hoc testing (p < .01) showed a significant difference between the hospitalized adolescents and the other two groups. A one-way ANOVA with the number of different words as a continuous variable showed a significant effect of group (*F* (57,2) = 8.43, p < .001, adjusted R^2^ = .20, post hoc *p* < .001). Hospitalized adolescents uttered fewer words and fewer different words than other adolescents. Thus, we see more similarity in the discourse of hospitalized adolescents.

### Textual Analysis

First, we conducted a correspondence analysis on the vocabulary in the entire corpus. Correspondence analysis is a technique for describing contingency tables (or crosstabs) and binary tables (“presence-absence” tables). It was introduced and systematically studied as a flexible technique for the exploratory analysis of multidimensional data by Benzécri (1973). The statistical unit used here is not the individual but the occurrence of a form. Columns constitute a partition of all adolescents’ life narratives and rows indicate the distribution of all the occurrences of forms (Lebart & Salem, 1994). Comparison of column profiles tells us about the proximity between the different categories created (in this case, pathology) and the vocabulary used.

**Figure 1:**
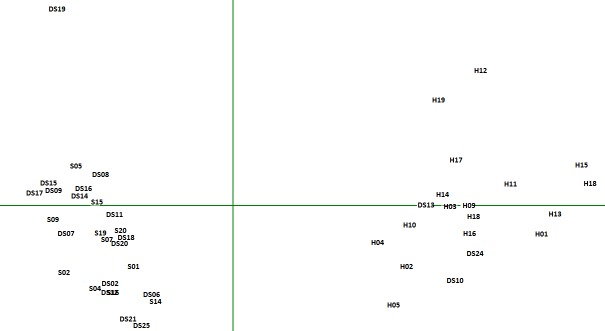
Correspondence analysis on vocabulary. H = Life narratives of hospitalized adolescents; DS = Life narratives of depressed adolescents attending school; S = Life narratives of non-depressed adolescents attending school.

A first correspondence analysis was performed on the entire corpus. Here, a section was created for each life narrative. Factorial designs allowed us to establish the proximity of life stories based on their vocabulary. This analysis considered 497 text units (words). The occurrence cut-off was set at 10. As shown in Figure 1, the first axis clearly opposes the life narratives of hospitalized depressed adolescents to those of adolescents in school (depressed or not). It highlights a specific vocabulary used by the hospitalized adolescents but not by the other groups. These results and the lexicometric characteristics show that the stories of adolescents attending school (depressed and not depressed) are somewhat similar, in terms of their size and of the vocabulary used. Hospitalized adolescents spoke less than the other groups and used fewer different words, but those words were more specific to their group.

To identify the specific words used by teenagers in their life stories, we conducted an analysis of specificity. Lafon (1980) studied the distribution of the frequency of a word in a corpus divided into several fragments. He proposed using the formulae for hypergeometric distribution, choosing the whole corpus as the norm for the fragments. These choices lead to the calculation of a probabilistic index that is valid for the whole range of frequencies. The calculation of this index for every form in the vocabulary enabled us to attribute to each fragment its own lexical specifications. The Hyperbase indexing program takes account of words with standard deviations greater than 2. If the standard deviation is large, the word is very specific to the class, that is, it is preferentially used by adolescents in this category.

The results presented in Table 2 showed that the specific words used by hospitalized adolescents revolve around their depression, their anxiety and their disease. Their feelings occupy an important place in their life stories (e.g., “I feel”). Our hypothesis that the pronoun “I” would be used more frequently was confirmed here, as we observed that depressive adolescent inpatients specifically used the pronouns “I” and “me,” in contrast to adolescents at school, whose stories were defined by the pronoun “we.” The non-depressed adolescents spoke about their experiences, their parents, the trips they had taken and their hobbies. Stories by the group of depressed adolescents at school were defined primarily by their education (e.g., studying, technical education) and focused on family problems (e.g., alcoholic father).

**Table 2:** Specificity of lemmas by group.

Hospitalized	σ	Depressed at school	σ	Non-depressed at school	σ

to feel	5.3	finally	6.6	we	8.3
daddy	4.3	here	7.1	experience	4.4
because	4.2	so	5.7	parent	3.9
depression	4.1	study	4.2	to meet	3.7
illness	3.5	technical	2.7	sport	3.6
me	3.3	now	3.4	to travel	2.9
anxiety	3	father	2.6	to go on	2.9
house	3	brother	2.5	hobby	2.7
I	3	alcoholic	2.4	activity	2.7

**Figure 2:**
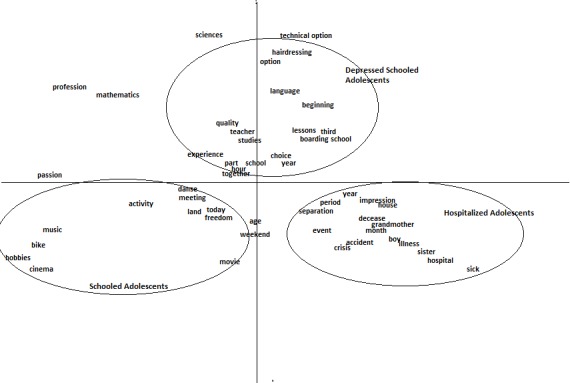
Correspondence analysis of the co-occurrences in vocabulary as a function of psychopathology.

A second correspondence analysis was performed on the co-occurrences in the life stories of adolescents in the three groups. A co-occurrence is the simultaneous presence of two forms in a text fragment. According to Mayaffre (2008), co-occurrences are the minimal forms of context. We can observe that, in non-depressed adolescents attending school, the topic of leisure is the most developed theme (Figure 2). We see co-occurrences of syntagmas such as *hobbies, movies*, and *dance*, while the concerns of the depressive adolescents attending school focused on their education, with co-occurrences including *option, teacher, professor, choice, education*, and *school year*. Co-occurrences for the group of hospitalized adolescents shed light on their relationships and feelings of loneliness (e.g., *father, mother, sister, friend*). Items such as *separation, illness, crisis, hospital, event*, and *death* are associated with these persons.

## Discussion

The aim of our study was to explore the life stories of depressive adolescents and compare them with non-clinical adolescents’ life stories. Our first analyses showed that hospitalized adolescents talked less and used significantly fewer different words. These results are consistent with the study by Pope et al. (1970), which found a slowdown in prosody and long break times (Marazziti et al., 2010). We also showed that depressed adolescents uttered fewer different words; that is, they spoke more about the same things. Indeed, a content analysis showed that these adolescents defined themselves primarily by their families and their personal problems. Many studies have described the impact of adverse life events predisposing individuals to develop both depressive and anxiety disorders (Spinhoven et al., 2010). Lack of family closeness, absence of family relationships (Bouma et al., 2008) or illness experienced by a close family member are all factors that may predispose to the occurrence of depression. In the life stories of depressed adolescents, family plays an important role because it is synonymous with difficulty, whereas the non-depressed adolescents move out from family life to include their peer group and friends in their stories. Thus, we can see that the depressed adolescents find it difficult to move forward, out of the domestic sphere. The disease also plays an important role in the stories of adolescent inpatients. Our results confirm the study by Clarke et al. (2006), which showed that depressed adolescents described themselves as depressed, sad or having low morale; the disease was part of their identity.

Through the study of the specific vocabulary used by our three groups, we were able to validate the hypothesis that adolescents with major depression would use “I” far more than other groups. These results are consistent with the observations of Rude, Gortner, and Pennebaker (1981) and Bucci and Freedman (2004). Hospitalized adolescents focus on themselves and their own role in what they are today. They are also more likely to use “I” while youths at school use impersonal sentences (e.g., “I have days when things are going well” (Hospitalized) vs. “There are days…” (School), “I have times when I feel good” (Hospitalized) vs. “There are times…” (School)). Our results are congruent with specific self-attribution in depressed inpatients. According to Habermas and Paha (2001), reasoning about one’s own development can be considered as a kind of complex self-evaluation that may be manifested in speech as a series of explicit assessments (Luborsky, 1993). These evaluations may take the form of verbs such as “I think” or “I see.” But we do not find these specific verbs in the discourse of depressed teenagers. In their subjectivity-punctuated sentences, we do not see what Erikson (1963) and Piaget (1965) described as the process of inferring meaning from past events, which is acquired in adolescence. Depressed adolescents present only factual descriptions of what happened to them.

In contrast, non-depressed adolescents use the personal pronouns “we” and “us,” which include the subject and the other. Adolescents at school do not feel alone in their definition of themselves. This group effect is reflected throughout their speech. They talk about their families, their friends, and their parents in the plural form (“My parents left me free to choose,” “My parents have never dictated to me,” “I have some respect for my parents”). Parents are presented in a positive light, as resources. The life stories of adolescents in school are strongly focused on the groups to which they belong and their hobbies. As shown by the co-occurrence analysis, adolescents define themselves in this way: “I like to laugh with my friends,” “I don’t hesitate to laugh a little,” “I dance and I enjoy it,” “I love to play sports” “Dance, I’m totally into it.” Their speech is punctuated by positive impressions (“I love,” “it’s fun,” “it’s great,” “awesome”).

Depressive adolescents attending school are characterized primarily by their concern with their education. Depressive adolescent students use significantly more conjunctions than other groups, such as “as,” because,” then,” and “but,” providing an explanation or comment about a life event. This specificity makes them more similar to the non-depressed adolescents. We observe an ability to decentralize the event – to step back and see the event’s contribution to their lives – that hospitalized depressed adolescents do not exhibit. Given the scarcity of references to family and friends compared to the other two groups, we can hypothesize that these depressed adolescents attending school have overinvested in the school sphere. They find it difficult to put things into perspective. To cope with difficulties in their lives, they focus on their academic achievements or their professional future, which they perceive as a cognitive investment under the form of options, courses, teachers, grades, but they do not see school as a social environment where they meet friends. These excessive concerns with academic success could eventually lead to anxiety in adolescents already suffering from depressive mood. Particularly in adolescence, anxiety and depressive disorders often occur as comorbid illnesses. Adolescents may focus on academic achievement but are unable to make the effort to succeed because of their symptoms (poor concentration, sleep disturbances, etc.). Many studies have also assessed the impact of depressive mood on academic achievement (Shahar et al., 2006; Da Fonseca et al., 2009; Jaycox et al., 2009). This contrasts with the concerns of non-depressed adolescents, for whom family and peers play a central role.

We also highlighted the importance of negative life events, specifically in the family (e.g., divorce, illness, grief). These results are consistent with those of Dalgard et al. (2006), who found an almost linear association between negative life events and depression among both girls and boys. This association becomes even stronger as the number of adverse events increases. As shown by statistical discourse analyses, the life stories of hospitalized adolescents focus primarily on the domestic sphere and problems at home. According to Barrera and Li (1996), even as adolescents develop a network of friendships and revise their parental relationships, the family retains a dominant position in terms of resources and attachment. If the family fails, the teenager’s well-being is threatened. Much research has also highlighted the role of the parenting, family and attachment in the development of depressive illness (Robertson & Simmons, 1989; Sheeber et al., 1997; Flett et al., 2012).

Depressed teenagers talk primarily about negative events in their families. Given that these past or present events have a heavy impact, further research is necessary to investigate the transition from depressed mood to depression and to longitudinally track the depressive spiral into which some adolescents are dragged. In light of our results, we can hypothesize that such a depressive spiral does exist. A teenager who is experiencing family problems (interpersonal difficulties or negative life events) focuses on academic achievement and is projected into a professional future. However, academic success may be hampered by depressive symptoms (Fröjd et al., 2008). Therefore, the teenager is no longer able to stay afloat; faced with family and school problems, he or she plunges into an even deeper depression.

These analyses allowed us to highlight specific themes narrated by each of the three groups of adolescents. Although life stories are personal and unique, analysis of such stories allows us to better understand the daily reality of depressive adolescents and the relationships between the life events they experience, daily stressors, depression and how they construct their personal history. The original feature of this research is the use of statistical analysis of textual data in clinical psychology. This technique allows us to analyze speech objectively and helps us deal with a large amount of data.

This exploratory study has also some limitations. It would be valuable to increase the number of stories in order to enrich the results. Work on the transcription of the corpus also seems necessary since we are working with an oral corpus which is subject to the subjectivity of the transcriber (especially with regard to punctuation). A future study should replicate the present findings. In addition, further analysis should be performed on the temporal coherence of the narratives according to group. Clinically, we did not take gender differences into account. Further work on a sample of adolescents attending school (Poudat & Boulard, 2014) shows that girls produce more family-oriented speech while boys produce speech more oriented towards the social sphere. These differences should be further explored with depressive psychopathology. Finally, combining the results of these analyses with quantitative data would be particularly interesting.

## Footnotes

A lemma is “essentially an abstract representation, subsuming all the formal lexical variations which may apply” (*Dictionary of Linguistics and Phonetics*, 2008).
